# Lack of Association Between Shape and Volume of Subcortical Brain Structures and Restless Legs Syndrome

**DOI:** 10.3389/fneur.2018.00355

**Published:** 2018-05-18

**Authors:** Marco Hermesdorf, Benedikt Sundermann, Rajesh Rawal, András Szentkirályi, Udo Dannlowski, Klaus Berger

**Affiliations:** ^1^Institute of Epidemiology and Social Medicine, University of Münster, Münster, Germany; ^2^Department of Clinical Radiology, University Hospital Münster, Münster, Germany; ^3^Department of Psychiatry, University of Münster, Münster, Germany

**Keywords:** restless legs syndrome, gray matter volume, subcortical brain structures, genetic risk, risk alleles

## Abstract

**Objective:**

Previous studies on patients with restless legs syndrome (RLS) yielded inconclusive results in the magnetic resonance imaging (MRI)-based analyses of alterations of subcortical structures in the brain. The aim of this study was to compare volumes as well as shapes of subcortical structures and the hippocampus between RLS cases and controls. Additionally, the associations between the genetic risks for RLS and subcortical volumes were investigated.

**Methods:**

We compared volumetric as well as shape differences assessed by 3 T MRI in the caudate nucleus, hippocampus, globus pallidus, putamen, and thalamus in 39 RLS cases versus 117 controls, nested within a population-based sample. In a subsample, we explored associations between known genetic risk markers for RLS and the volumes of the subcortical structures and the hippocampus.

**Results:**

No significant differences between RLS cases and controls in subcortical and hippocampal shapes and volumes were observed. Furthermore, the genetic risk for RLS was unrelated to any alterations of subcortical and hippocampal gray matter volume.

**Interpretation:**

We conclude that neither RLS nor the genetic risk for the disease give rise to changes in hippocampal and subcortical shapes and gray matter volumes.

## Introduction

Restless legs syndrome (RLS) is a sensorimotor disorder affecting 2.5–10% of the general population (1). RLS is characterized by unpleasant sensations in the legs or other extremities combined with an urge to move in order to reduce the discomforting sensations. These symptoms typically worsen during periods of rest, thus having a negative impact on sleep and quality of life (2, 3). Genetic factors play an important role in RLS as it has been revealed that several single nucleotide polymorphisms (SNPs) contribute to the development of the disease (4, 5). Furthermore, RLS is believed to be a result of iron insufficiency in the brain, presumably caused by improper iron transportation across the blood–brain barrier leading to dysregulated dopaminergic neurotransmission (6).

Due to the specific role of subcortical structures in dopaminergic neurotransmission (7) and their role in iron deposition and motor function (8), these structures are of particular interest in the search for neurobiological correlates of RLS. Previous studies employing magnetic resonance imaging (MRI) of the brain provided conflicting results regarding volumetric changes of subcortical gray matter in RLS cases. In particular, a reduction in gray matter volume has been observed in the left hippocampus (9), while others found a significant increase in left hippocampal gray matter associated with RLS (10). Increased gray matter volume in the pulvinar nuclei located inside the thalamus has also been reported (11). In contrast, several studies found no significant associations between RLS and alterations of subcortical gray matter volume (12–15). Most of these studies applied voxel-based morphometry for the detection of local changes in gray matter volume across the brain (9–14). However, specific methods have been developed to detect localized shape differences in subcortical regions and the hippocampus, considering the specific signal characteristics of these brain structures (16). Only a single study investigated such localized shape differences of the thalamus, but did not detect significant shape differences in patients with RLS versus controls (15). Localized shape differences in subcortical regions other than the thalamus have not been investigated in patients with RLS.

The present study aimed to contrast potential differences in localized shape and overall volume of several subcortical gray matter structures (caudate nucleus, globus pallidus, putamen, and thalamus) as well as the hippocampus between RLS cases and controls, all participants in the BiDirect Study. Additionally, we investigated associations between known genetic risks for RLS and potential alterations of subcortical gray matter volume, since MRI-detectable changes in these subcortical structures might be a mediator in the pathway between genotype and RLS.

## Materials and Methods

### Participants

**Figure 1:**
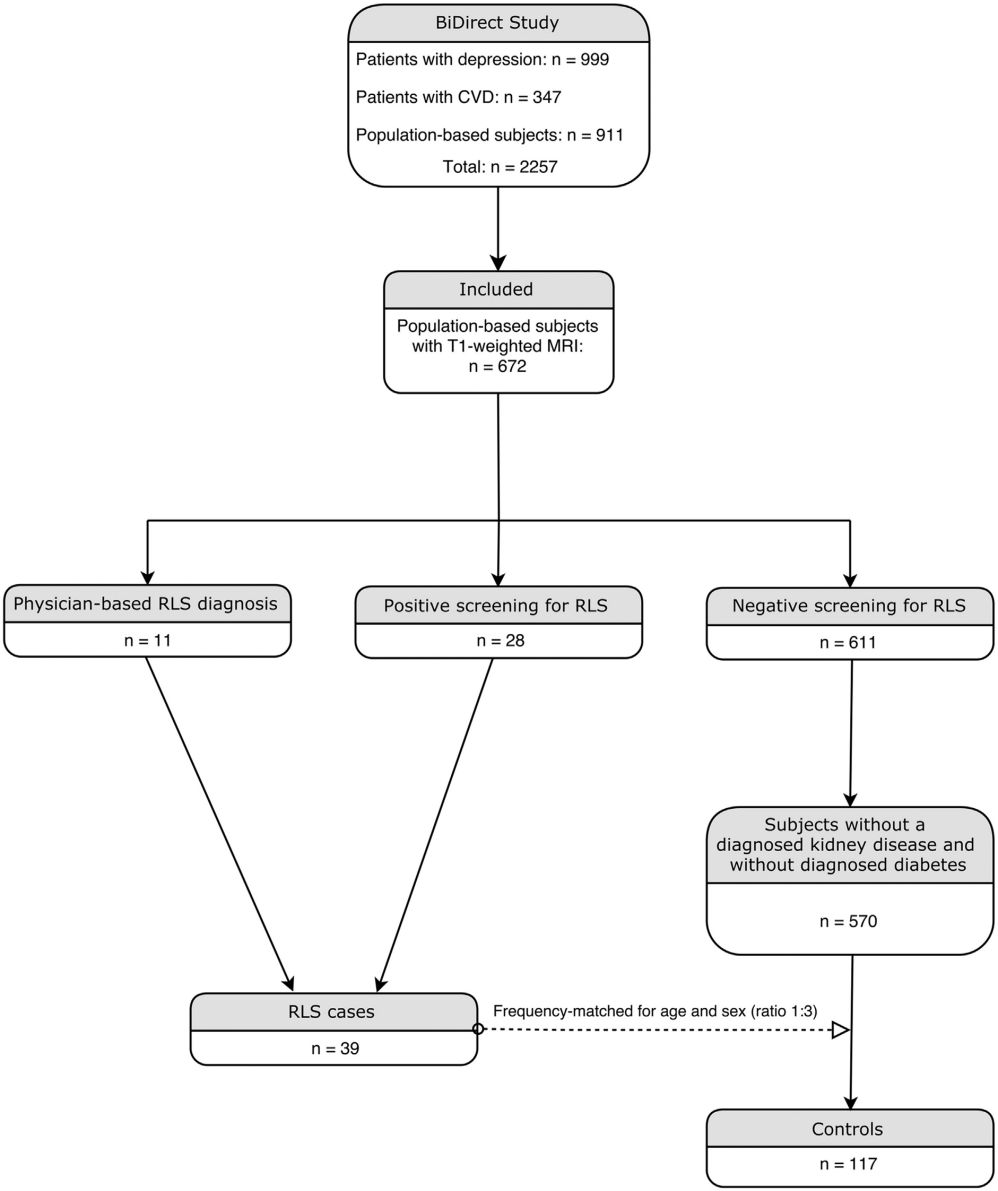
Flowchart illustrating the inclusion procedure.

The ongoing BiDirect study is conducted to investigate associations between subclinical arteriosclerosis and depression. For this purpose, the BiDirect study integrates two patient cohorts, one including patients with depression, the other patients with cardiovascular disease, and one general population cohort into one project. Details on methods and design of the BiDirect Study are provided elsewhere (17, 18). Participants in the general population cohort were randomly sampled from the population register of the city of Münster, resulting in 911 individuals included in this cohort. All participants had to be in the age range from 35 to 65 years at recruitment. Informed consent was signed by all study participants in the BiDirect project, which was approved by the ethics committee of the University of Münster and the Westphalian Chamber of Physicians. Within the general population cohort, we performed a nested case-control analysis. Participants from the two patient cohorts of the BiDirect Study were thus not considered in the present analysis. Participants who did not undergo T1-weighted MRI were excluded and RLS-status was assessed in face-to-face interviews by a set of questions that were based on the criteria established by the International RLS Study Group (19). This question set has previously been validated against a standardized neurological examination and both were in good agreement (20). In addition, a physician diagnosis of RLS in the past was assessed. Study participants who positively answered questions on all minimum criteria or reported a physician diagnosis of RLS were classified as RLS cases. In total, 11 participants had a prior physician-based diagnosis of RLS and 28 participants were screened positive by the question set. Participants without a physician diagnosis of RLS and a negative screening were classified as controls. Controls with a previously diagnosed kidney disease and/or diabetes were excluded from the analysis. Based on the group of RLS cases, controls were frequency-matched one to three by the variables age and sex. This resulted in 39 RLS cases and 117 controls for the nested case-control analysis as depicted in Figure 1. Furthermore, we conducted a sensitivity power analysis using G*Power (21), revealing that we can detect substantial effects (*f* = 0.29) with a power of 95% in the shape analysis.

### Image Acquisition

Magnetic resonance imaging of the brain was performed in all BiDirect participants without contraindications. Structural 3D T1-weighted turbo field echo imaging was performed on a 3 T scanner (Intera, Philips, Best, Netherlands) to obtain 160 sagittal slices with a thickness of 2 mm (reconstructed to 1 mm), resulting in a voxel-size of 1 × 1 × 1 mm (TR = 7.26 ms, TE = 3.56 ms, 9° flip angle, matrix dimension 256 × 256, FOV = 256 × 256 mm).

### Image Preprocessing

Magnetic resonance imaging data were preprocessed using FSL (22) version 5.06. Images were linearly registered to the MNI152 template using FLIRT (23). If necessary, images were cropped or bias-field corrected with fsl_anat^1^ to ensure optimal registration. The inverse transformation matrix was then applied to the predefined subcortical shape models provided by FSL. With these predefined shape models in native space, subcortical structures of interest (caudate nucleus, hippocampus, globus pallidus, putamen, and thalamus) were segmented from the participants’ native space images using a Bayesian appearance model in FIRST (16) and modeled as surface meshes. In a last step, the subcortical surfaces were aligned to a sample-specific mean shape of the respective surface structures applying a 6 degree of freedom transformation whereby differences in rotation and translation were removed.

For the purpose of a volume-based analysis, subcortical structures were boundary corrected and the respective volumes of interest were extracted. In order to adjust for the overall brain volume in the course of analyses, brain volume (i.e., gray and white matter) was estimated by partial volume estimation in FAST (24).

### Genotyping

Genotyping was conducted using the Illumina PsychChip array (Illumina, San Diego, CA, USA). Several SNPs in *MEIS1, BTBD9, MAP2K5, PTPRD*, and *TOX3/BC034767* (4, 25–27) have previously been associated with RLS and were selected for the study at hand. Imputation was performed using IMPUTE version 2.3.2 (28). SNPs being in linkage disequilibrium (*R*^2^ ≥ 0.8) or with a minor allele frequency below 5% were excluded from the analysis. Statistics regarding linkage disequilibrium were derived from the database of the Broad Institute.^2^

### Comorbidities

We assessed comorbidities using data from face-to-face interviews as well as laboratory data. Previous physician-based diagnoses of stroke, myocardial infarction, and cancer were collected by self-report. Participants were classified as hypertensive if the mean of the second and third blood pressure readings for systolic blood pressure was ≥140 mm Hg or the diastolic blood pressure exceeded 89 mm Hg. Furthermore, participants with a self-reported physician-based diagnosis of hypertension in combination with use of antihypertensive medication according to the Anatomical Therapeutic Chemical (ATC) Classification System (ATC C02A, C02D, C02L, C03, C07, C08, C09) were also defined as having hypertension. Depression was assessed as a previous physician-based diagnosis *via* self-report or if participants scored ≥16 points on the Center for Epidemiologic Studies Depression Scale (29). Body size and weight were assessed and participants with a body mass index larger than 30 $\frac{\text{kg}}{m^{2}}$ were classified as obese. The presence of thyroid disease was assessed by self-report of a physician-based diagnosis or intake of relevant medication (ATC H03). Thyroid-stimulating hormone (TSH) and free thyroxine (fT_4_) levels were used to estimate hypothyroidism (TSH > 4.8 $\frac{\mu \text{IU}}{\text{mL}}$ and fT_4_ < 13 $\frac{\text{pmol}}{\text{L}}$) as well as hyperthyroidism (TSH < 0.3 $\frac{\mu \text{IU}}{\text{mL}}$ and fT_4_ > 23 $\frac{\text{pmol}}{\text{L}}$) and participants in either category were also defined as having thyroid disease. Migraine was assessed as physician-based diagnosis *via* self-report or current use of relevant medication (ATC N02CA, N02CC). A comorbidity index was calculated by summing up the presence of the previously described conditions (stroke, myocardial infarction, cancer, hypertension, depression, obesity, thyroid disease, and migraine). A similar index of cumulative disease burden has been used previously in the context of RLS (30).

### Statistical Analysis

Participants with RLS and controls were compared on orthogonal displacements at each vertex regarding the sample-specific mean surfaces of the subcortical structures of interest. These analyses were conducted with a cluster-based *F*-test implemented in FSL randomize (31) with 5,000 permutations. Statistical threshold for significance was set to *p* < 0.05. Extracted volumes of the subcortical structures were compared across groups by several analyses of covariance (ANCOVAs) while adjusting for overall brain volume. The obtained *p*-values were corrected for false discovery rate (FDR) following the Benjamini–Hochberg procedure (32).

Genotyping data were available for 137 participants. For each participant, the number of risk alleles per SNP was noted. For each respective SNP, a logistic regression was conducted with RLS as dependent variable and risk allele frequency as predictor along with age and sex as covariates of no interest. A weighted genetic risk score (GRS) was calculated for each SNP by multiplying the risk allele frequency of the respective SNP with the odds ratio obtained by the logistic regression. Each respective GRS was used as a predictor in multiple regression analyses with the subcortical brain volumes as dependent variables while adjusting for age, sex, and overall brain volume. The analyses of extracted subcortical volumes and genotyping data were conducted in SPSS version 22 (IBM, Armonk, NY, USA).

## Results

### Subject Demographics

A comparison of group characteristics is summarized in Table 1. Participants with RLS and controls did not differ in terms of age and sex. Distributions of comorbidity load were significantly different across groups and the median comorbidity load was higher in RLS cases. Genotyping data were available for 137 (87.8%) participants. The remaining 19 participants were thus not considered for the analyses of genotyping data.

**Table 1:** Comparison of demographic characteristics across groups.

Variable	Participants with RLS *n* = 39	Controls *n* = 117	*p*
Age: mean (SD)	51.21 (8.51)	51.29 (8.33)	0.957
Women: *n* (%)	26 (66.7%)	78 (66.7%)	1
Comorbidity index^a^: median (IQR)	2 (1–2)	1 (0–2)	<0.01
Genotyping data available: *n* (%)	35 (89.7%)	102 (87.2%)	
Duration of disease in years^a^: median (IQR)	5 (2.5–10)		
**Frequency of symptoms: *n* (%)**			
Less than once in per month	5 (12.8%)		
1–3 times per month	11 (28.2%)		
1–2 times per week	6 (15.4%)		
3–6 times per week	6 (15.4%)		
Daily	8 (20.5%)		
Undetermined	3 (7.7%)		

*RLS, restless legs syndrome; IQR, interquartile range*.

*^a^One participant had incomplete comorbidity data and two participants had missing data on disease duration*.

*Age was compared with a t-test. Distribution of sex was compared with a χ^2^ test and comorbidity load with a Mann–Whitney U-test*.

### Shape Analyses

**Figure 2:**
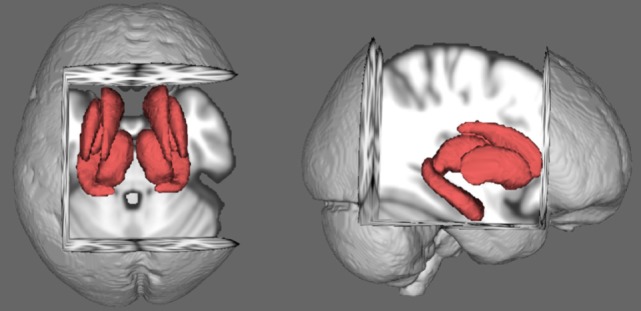
Average shapes of the rigidly aligned subcortical structures in the sample.

The comparisons of the shapes in the caudate nucleus, hippocampus, globus pallidus, putamen, and thalamus across groups did not yield significant differences in either hemisphere. The subcortical and hippocampal shapes of the sample are shown in Figure 2. The analyses of extracted volumetric data did not reveal significant group differences after FDR correction (Table 2).

**Table 2:** Comparison of subcortical volumes (mm^3^ and SE) across groups.^a^

Region	RLS cases *n* = 39	Controls *n* = 117	*p*	FDR-sig
Left nucleus caudate	3,280.88 (58.56)	3,214.5 (33.8)	0.328	n.s.
Left hippocampus	3,881.74 (56.85)	4,012.67 (32.82)	0.048	n.s.
Left globus pallidus	1,538.77 (44.81)	1,629.51 (25.86)	0.082	n.s.
Left putamen	4,786.91 (81.31)	4,854.70 (46.93)	0.471	n.s.
Left thalamus	7,481.04 (78.46)	7,542.56 (45.29)	0.498	n.s.
Right nucleus caudate	3,381.60 (56.37)	3,308.45 (32.54)	0.263	n.s.
Right hippocampus	4,012.95 (61.09)	4,075.04 (35.26)	0.380	n.s.
Right globus pallidus	1,629.77 (39.02)	1,688.05 (22.52)	0.198	n.s.
Right putamen	4,811.87 (70.33)	4,886.11 (40.59)	0.362	n.s.
Right thalamus	7,252.85 (76.20)	7,270.17 (40.59)	0.844	n.s.

*RLS, restless legs syndrome; FDR-sig, false discovery rate corrected significance at q < 0.05; n.s., not significant*.

*^a^The group comparison is adjusted for overall brain volume*.

### Associations Between Risk allele Frequency and RLS

The logistic regressions yielded significant associations between RLS and the risk allele (G) frequency in rs11635424, located in *MAP2K5*. Due to the small sample size, no significant associations were found for the remaining eight SNPs. However, the magnitude of the odds ratios indicated a higher risk for RLS concerning the majority of the SNPs (Table 3), in line with prior reports (4, 25, 27, 33).

**Table 3:** Associations between allele frequency and RLS.^a^

Single nucleotide polymorphisms (risk allele)	Odds ratio	*p*
rs12469063 (G)	1.403	0.252
rs6710341 (G)	1.029	0.940
rs3923809 (A)	1.031	0.917
rs4714156 (C)	1.395	0.366
rs9394492 (C)	0.945	0.848
rs4626664 (A)	1.339	0.455
rs11635424 (G)	2.149	0.032
rs6747972 (G)	1.049	0.852
rs3104767 (G)	1.472	0.197

*RLS, restless legs syndrome*.

*^a^The logistic regressions are adjusted for age and sex*.

### Genetic Risk for RLS and Subcortical Volumes

No significant associations between the odds ratio weighted GRS derived from the respective SNPs and subcortical as well as hippocampal volumes were found. The regression coefficients are presented in Table 4.

**Table 4:** Regression coefficients for the GRS per subcortical region.

	GRS rs12469063	GRS rs6710341	GRS rs3923809	GRS rs4714156	GRS rs9394492	GRS rs4626664	GRS rs11635424	GRS rs6747972	GRS rs3104767
Left caudate nucleus	22.17	59.96	18.86	−21.91	4.56	82.02	−3.489	−21.52	3.802
Left hippocampus	29.36	9.56	80.71	49.67	37.60	46.21	−33.49	11.85	14.77
Left globus pallidus	31.40	2.24	−20.92	−29.71	9.74	34.41	11.43	−17.63	−7.20
Left putamen	3.02	65.17	−62.88	−78.58	−52.31	41.77	35.82	−4.80	−20.85
Left thalamus	−14.97	20.83	−29.97	−62.61	−48.98	−10.72	−37.39	−25.45	74.51
Right caudate nucleus	−6.84	38.38	55.30	3.12	−20.29	63.33	−0.13	−13.95	−11.49
Right hippocampus	−9.53	48.50	−14.32	−15.16	48.69	22.36	−28.04	−65.148	28.52
Right globus pallidus	34.81	34.24	−17.79	−14	26.33	44.08	−2.37	4.56	−13.61
Right putamen	−14.59	64.07	17.27	−32.39	−52.04	40.80	−20.83	−8.14	−0.66
Right thalamus	1.60	28.61	−20.06	−67.19	−17.31	37.72	−40.70	−14.14	72.87

*GRS, genetic risk score*.

*The regression analyses were adjusted for age, sex, and overall brain volume*.

## Discussion

In this nested case-control study, we examined potential alterations in shape and volume of subcortical structures and the hippocampus in cases with RLS versus controls. While potential volumetric alterations of subcortical structures and the hippocampus have been investigated previously using VBM (9–15), shape differences in the caudate nucleus, hippocampus, globus pallidus, and putamen have not been compared before between cases with RLS and controls. Analyzing shape differences, however, is important in order to determine the exact locations where potential anatomical changes in subcortical structures occur. Knowledge of localized shape differences may also aid the interpretation of the relationship with other anatomical findings, e.g., when localized changes in thalamic shape are associated with adjacent reductions of white matter volume (34). Our analyses revealed no group differences in either shape or volume of the caudate nucleus, hippocampus, globus pallidus, putamen, and thalamus. The lack of volume differences supports previous findings (12–15), suggesting that RLS is not accompanied by any changes of subcortical gray matter. Instead, it seems more likely that alterations of the dopaminergic system (6), possibly induced by genes involved in neurodevelopment [*MEIS1* (35, 36) and *TOX3* (37)], protection of dopaminergic neurons [*MAP2K5* (38)], sleep disturbances [*BTBD9* (39)], modulation of dopaminergic neurotransmission [*PTPRD* (40)], and iron regulation within the brain [*BTBD9* (41)], may lead to changes in functional brain networks. In particular, increased functional connectivity has been reported in sensory-thalamic, basal ganglia-thalamic, and other cortical and subcortical networks in patients with RLS, whereas symptom severity correlated with increased network connectivity (42). Hence, in the absence of gray matter alterations, RLS is more likely to be characterized by inefficient network performance.

Although RLS has previously been associated with several SNPs within regions of the above-mentioned genes (4), the exact mechanisms how these SNPs contribute to the development of RLS are still unknown. Hence, we also explored potential associations between known genetic risk markers for RLS and alterations of subcortical volumes to evaluate if these are a potential mediator of the genotype-disease association. Only SNP rs11635424 was significantly associated with RLS. While most of the remaining RLS-related SNPs indicated risks, i.e., odds ratios larger than 1 for the risk alleles, these associations did not reach statistical significance given the small sample size in our study. The magnitude of effect sizes is largely in line with previous studies (4, 25, 27, 33), suggesting that larger samples are advantageous to detect effects of allele frequency in the context of RLS. With regards to the volume of the subcortical structures and the hippocampus, we did not find a significant association with SNP rs11635424 or any of the other eight SNPs, suggesting that RLS-related variations in the genome do not play an important part in the volumetric appearance of subcortical structures and the hippocampus.

The present study is limited by its sample size which is rather small regarding the search for genetic factors contributing to the development of RLS. However, the primary aim was to compare subcortical as well as hippocampal shapes and volumes between RLS cases and controls and to analyze the influence of the odds ratio weighted genetic risk for RLS on subcortical and hippocampal volumes. Within the field of 3 T MRI-literature, the present study is the largest investigating potential volumetric alterations in RLS cases versus controls.

We conclude that RLS is unrelated to changes in shape and volume of the caudate nucleus, hippocampus, globus pallidus, putamen, and thalamus. The SNP rs11635424 was significantly associated with RLS in our sample. The odds ratio weighted GRS from each of the nine SNPs as well as a summed GRS do not account for any volume alterations of subcortical gray matter.

## Ethics Statement

This study was carried out in accordance with the recommendations of the ethics committee of the University of Münster and the Westphalian Chamber of Physicians with written informed consent from all subjects. All subjects gave written informed consent in accordance with the Declaration of Helsinki. The protocol was approved by the University of Münster and the Westphalian Chamber of Physicians.

## Author Contributions

Design and concept of the BiDirect Study: KB. Preprocessing and data analyses: MH, RR, and AS. Drafting the manuscript and figures: MH. Technical assistance and commenting on the preprocessing of imaging data: BS and UD.

## Conflict of Interest Statement

MH, BS, RR, AS, and UD have no conflicts of interest to disclose. For the conduction (2007–2014) of a study on the course of restless legs syndrome, KB has received unrestricted grants to the University of Münster from the German Restless Legs Society and Boehringer Ingelheim Pharma, Mundipharma Research, Neurobiotec, Roche Pharma, UCB Germany, UCB Switzerland, and Vifor Pharma.

## Footnotes

^1^http://fsl.fmrib.ox.ac.uk/fsl/fslwiki/fsl_anat (Accessed: 2017).

^2^https://archive.broadinstitute.org/mpg/snap (Accessed: 2017).
